# Cocirculation of endemic and recently introduced West Nile Virus lineage 1 clades in Southern Spain

**DOI:** 10.1016/j.onehlt.2026.101368

**Published:** 2026-02-18

**Authors:** Carlos S. Casimiro-Soriguer, Maria Lara, Irene Pedrosa-Corral, Cristina Gómez-Camarasa, Nicola Lorusso, J. Alberto Chaves, Jose M. Navarro-Marí, Laura Merino, Jose A. Lepe, Joaquin Dopazo, Sara Sanbonmatsu-Gámez, Javier Perez-Florido

**Affiliations:** a Platform of Computational Medicine, Fundación Progreso y Salud (FPS), Hospital Virgen del Rocio, 41013 Sevilla, Spain; b Institute of Biomedicine of Seville, IBiS, University Hospital Virgen del Rocío/CSIC/University of Sevilla, 41013 Sevilla, Spain; c Laboratorio de Referencia de Virus de Andalucía, Servicio de Microbiología, Hospital Virgen de las Nieves, 18014 Granada, Spain; d Instituto de investigación biosanitaria, ibs.GRANADA, 18012 Granada, Spain; e Dirección General de Salud Pública y Ordenación Farmacéutica, Consejería de Salud y Consumo, Junta de Andalucía, 41020 Sevilla, Spain; f Preventive Medicine and Public Health, Maimonides Biomedical Research Institute (Imibic), Córdoba, Spain; g Servicio de Microbiología, Unidad Clínica Enfermedades Infecciosas, Microbiología y Medicina Preventiva, Hospital Universitario Virgen del Rocío, 41013 Sevilla, Spain; h Centro de Investigación Biomédica en Red en Enfermedades Infecciosas (CIBERINFEC), ISCIII, Madrid, Spain

**Keywords:** West Nile virus, Genomic surveillance, Lineage 1 clades, Phylogenetics, Outbreak, One Health

## Abstract

In 2024, Andalusia (Southern Spain) reported its largest recent West Nile virus (WNV) outbreak, with 106 confirmed human cases and 16 deaths. Genomic analysis of 27 WNV isolates revealed the co-circulation of multiple WNV lineage 1 clades, including both endemic and recently introduced strains, reflecting a marked increase in viral genetic diversity. These findings underscore the critical role of integrated genomic surveillance systems in monitoring pathogen evolution and supporting timely public health responses to emerging zoonotic threats.

## Highlights

• Genomic analysis of 27 WNV isolates reveals the co-circulation of endemic and newly introduced lineage 1 clades in Spain.
• Phylogenetic analysis identifies persistent WNV strains from previous outbreaks alongside novel variants.
• The findings highlight Spain's role in the circulation of WNV between Africa and Europe.
• Findings also underscore the need for enhanced One Health surveillance in Spain.

## 1 Introduction

WNV has been detected in mainland Europe since the mid-1990s [1] with two circulating lineages (1 and 2) causing sporadic outbreaks, affecting birds [2], horses [3] and humans [4]. In particular, the first occurrences in southern Spain were reported in early 2000s, with notable outbreaks occurring in Andalusia in 2010 and 2020 [5]. Both, lineage 1 and, more recently, lineage 2 have been detected in the region [5], [6]. The 2020 outbreak marked a turning point for regional surveillance efforts. However, in 2024, a resurgence occurred, resulting in the largest WNV outbreak in Andalusia to date, with 106 confirmed human cases and 16 fatalities [6], which prompted the inclusion of WNV in the genomic surveillance circuit of Andalusia [7], [8]. Here we provide a detailed molecular characterization of the last WNV lineage 1 outbreak in Andalusia, documenting the coexistence of imported viruses along with endemic viruses from a phylogenetic clade present in the south of Spain at least since 2020.

## 2 Genomic surveillance circuit in Southern Spain (Andalusia)

### 2.1 Sample collection in the surveillance circuit

Epidemiological surveillance in Andalusia is coordinated through the “Sistema de Vigilancia Epidemiológica de Andalucía” (SVEA) [9], integrated within the national surveillance network and guided by a One Health approach. The system combines human, animal, entomological, and environmental data streams to enable early detection and coordinated response to zoonotic threats like WNV. Surveillance activities follow EU case definitions [10] and involve routine case reporting, laboratory confirmation, and integration of data from equine and wildlife surveillance, supported by sectoral collaboration across regional ministries [11]. In response to previous outbreaks, Andalusia implemented an Integrated Surveillance and Vector Control Programme that mandates entomological monitoring and local mosquito control plans in affected municipalities [12]. The surveillance infrastructure is reinforced by genomic data integration through the regional “Sistema Integrado de Epidemiología Genómica de Andalucía” (SIEGA) platform, enabling pathogen lineage tracking to inform risk assessments and response strategies [8].

Specimens, including whole blood and urine, collected from cases with clinical suspicion of WNV infection in Andalusia (Southern Spain) during 2024 (see Table 1) were submitted to the Andalusian Virus Reference Laboratory Samples following the SVEA Protocol for WNV surveillance and alert [9].

**Table 1:** WNV isolates from human reported in this study with its corresponding sample source, date and location (province), along with the average sequencing depth and genome coverage. These sequences are represented in Fig. 1 along with the sequences from other isolates from human, mosquitoes, birds and horses.

ENA ID	Source	Collection date	Location	Mean depth	Genome Coverage
ERS25346490	Urine	17/08/2021	Sevilla	5315.63	99.28
ERS25346491	Urine	03/09/2021	Sevilla	4476.02	88.79
ERS25346492	Urine	21/09/2021	Sevilla	1596.51	97.90
ERS25339721	Urine	11/07/2024	Sevilla	5518.87	97.95
ERS25339711	Urine	14/07/2024	Sevilla	4127.72	99.49
ERS25339715	Urine	17/07/2024	Sevilla	3213.48	98.16
ERS25339713	Urine	22/07/2024	Sevilla	2040.80	99.28
ERS25339712	Blood	22/07/2024	Sevilla	19,197.25	95.29
ERS25339707	Urine	31/07/2024	Sevilla	2533.45	99.28
ERS25339722	Urine	03/08/2024	Sevilla	2421.36	99.28
ERS25339714	Urine	03/08/2024	Sevilla	3877.46	98.16
ERS25339719	Urine	07/08/2024	Sevilla	3606.40	97.95
ERS25339717	Urine	07/08/2024	Sevilla	2998.61	97.95
ERS25339718	Urine	12/08/2024	Sevilla	3724.15	97.72
ERS25339725	Urine	17/08/2024	Sevilla	3883.71	97.79
ERS25339710	Urine	24/08/2024	Sevilla	5296.19	98.16
ERS25339716	Urine	27/08/2024	Sevilla	3555.52	97.95
ERS25339727	Urine	30/08/2024	Sevilla	7783.96	95.59
ERS25339724	Urine	01/09/2024	Sevilla	9695.63	98.16
ERS25339728	Urine	04/09/2024	Cordoba	7763.31	98.16
ERS25339708	Blood	04/09/2024	Cordoba	7914.82	99.49
ERS25339723	Urine	06/09/2024	Sevilla	5448.10	61.18
ERS25339720	Urine	10/09/2024	Jaen	8986.46	98.16
ERS25339709	Urine	11/09/2024	Sevilla	14,155.03	60.37
ERS25339705	Urine	12/09/2024	Huelva	11,946.43	99.28
ERS25339706	Urine	18/09/2024	Cordoba	15,628.23	95.57
ERS25339726	Blood	20/09/2024	Sevilla	28,404.83	97.28

### 2.2 Sample sequencing

RNA was extracted using the QIAsymphony DSP Virus/Pathogen Mini Kit (Qiagen, Hilden, Germany). A qRT-PCR targeting the 3’UTR region of the WNV genome was employed to detect viral RNA [13] in urine, serum and whole blood samples.

RNA extracted from WNV positive samples was reverse transcribed using LunaScript® RT SuperMix Kit (New England Biolabs, Ipswich, MA, USA). Multiplex PCR was carried out using Q5® Hot Start High-Fidelity (New England Biolab) protocol, utilizing a panel of 41 primer pairs previously validated for WNV genome amplification [5], [14].

PCR products were purified with Agencourt AMPure XP beads (Beckman Coulter), and libraries were prepared using the Illumina DNA Prep Kit. Samples were pooled in equal concentrations after quantification by Qubit 4 fluorometer (Invitrogen). Sequencing was performed on the iSeq 100 System using a iSeq 100 i1 Reagent v2 (300-cycle) (Illumina).

Raw paired-end reads (150 bp × 2) were analyzed using the nf-core/viralrecon pipeline software (v.2.6.0) [15] (see also [5], [6]). Quality control, trimming, alignment to a WNV lineage 1 reference genome (JF719067), and variant calling were performed using standard tools including *Bowtie 2* (v2.4.4) [16], iVar (v1.4) [17] and *bcftools* (v1.16) [18]. A consensus genome was generated for each sample based on high-confidence variants (allele frequency ≥ 0.75).

The new WNV sequences reported here are available in the European Nucleotide Archive (ENA) database under the project identifier PRJEB43037.

**Fig. 1:**
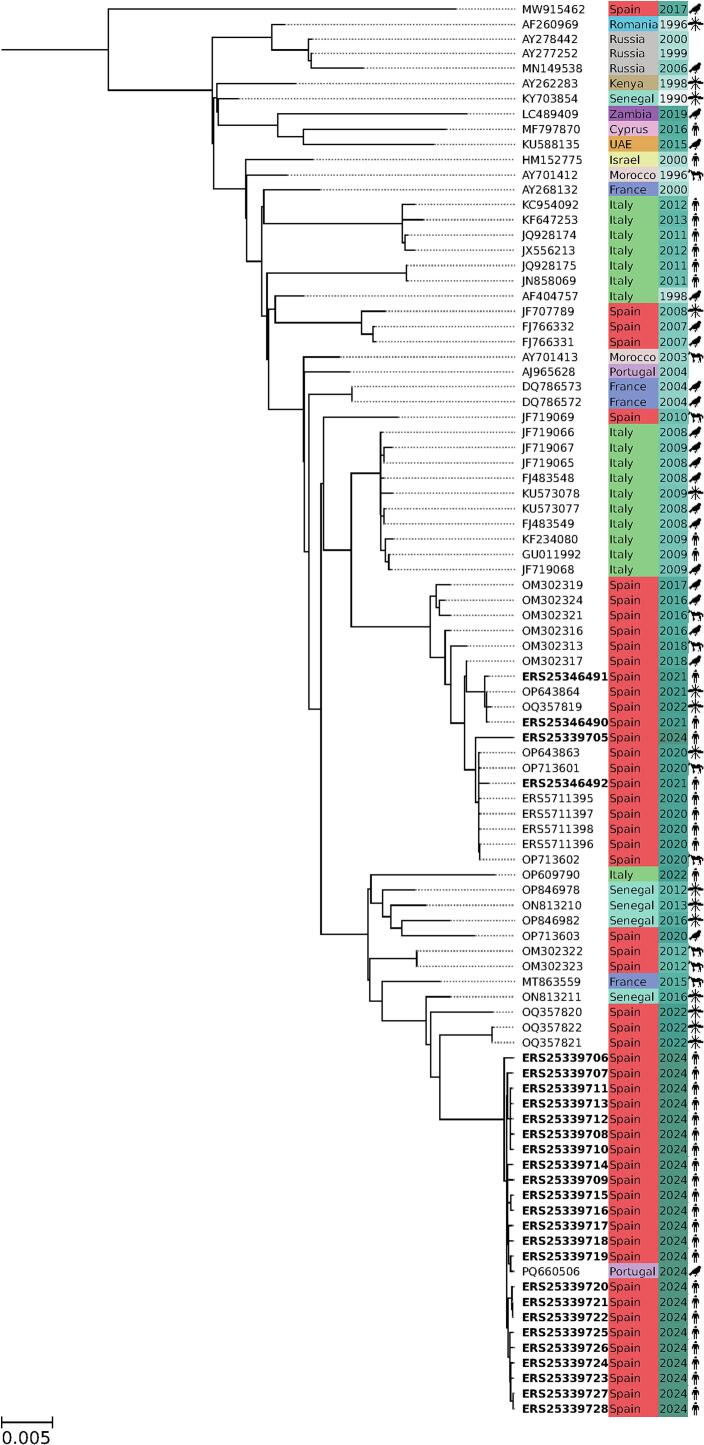
Phylogenetic tree of the current Spanish samples in the context of the recent phylogenetically-related WNV sequences available (See Table 1 and Supplementary Table 1). Phylogenetic tree of the current Spanish samples in the context of the recent phylogenetically-related WNV sequences available (See Table 1 and Supplementary Table 1).

### 2.3 Phylogenetic analysis of the 2024 outbreak in Andalusia

A set of 228 whole genomes of WNV lineage 1 from previous outbreaks found in the GenBank repository (listed in Supplementary Table 1) were downloaded and aligned with the 24 new whole genome sequences from the 2024 outbreak, plus 3 extra whole genomes from the 2021 outbreak, reported in this study (Table 1) using MAFFT v7.515 [19].

**Supplementary Fig. S1:**
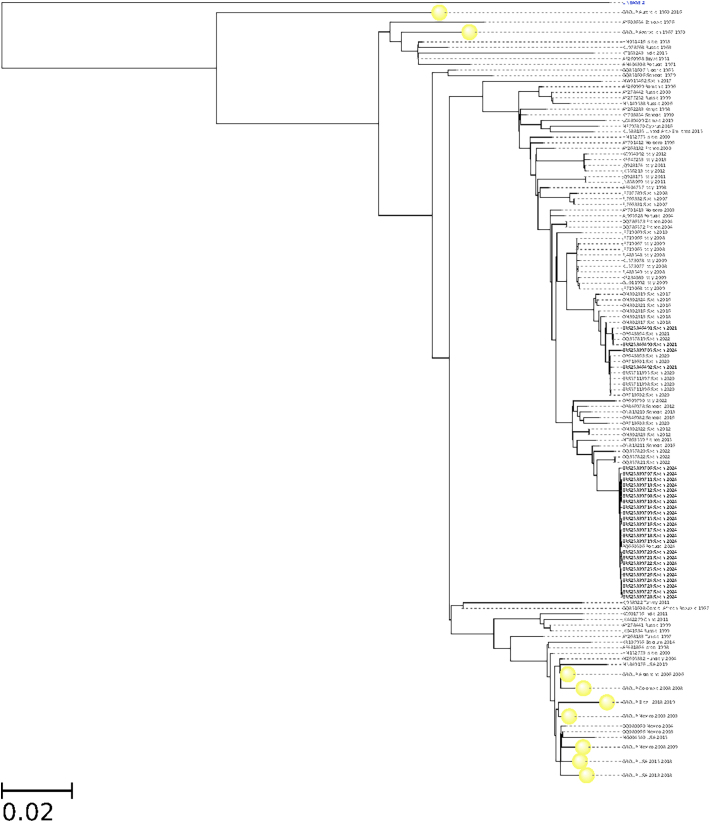
Phylogenetic tree of 228 whole genomes of WNV lineage 1 from previous outbreaks found in the GenBank repository (See also Supplementary Table 1).

Fig. 1 shows a phylogenetic tree depicting the current Spanish samples in the context of the recent phylogenetically-related WNV sequences available, reconstructed using the Augur toolkit (v21.0.0) [20]. This phylogeny can be interactively visualized using the SIEGA Nextstrain server [21]. Finally, Supplementary Fig. S1 depicts an expanded phylogenetic tree, constructed with a total of 255 whole genome sequences, covering virtually all WNV lineage 1 sequences available in the public repositories, that portraits the phylogenetic origin of all the European samples in the context of the WNV sequences reported worldwide.

## 3 Discussion

Fig. 1 provides a clear picture of the recent 2024 outbreak in the context of all the data available on recent WNV lineage 1 outbreaks. Largely, the 2024 outbreak clade, which also includes one Portuguese isolate (PQ660506) dating also from 2024 [22], derives from previous samples from mosquito reported in 2022 in Spain (OQ357821, OQ357822 and OQ357820) [23]. All these sequences derive from previous samples reported in 2020 in birds (OP713603), demonstrating the key role of Spain in the traffic of WNV between Europe and Africa [24], as confirmed by other related sequences from Senegal (OP846978 and ON813210 in 2013 as well as OP846982 and ON813211 in 2016), France (MT863559 in 2015) and Italy (OP609790 in 2022) Data on bird's migration [25] confirm that Spain is the transit point used for travelling between Africa and Europe. All the infected subjects in Table 1 came from small villages close to the Guadalquivir river and swamps around.

Interestingly, one of the sequences from the recent 2024 outbreak (See ERS25339705 in Table 1) is not related to the rest of isolates of the current outbreak reported here but it derives from WNV from the 2020 and 2021 outbreaks [5]. This clade has been continuously present in Spain at least since 2016, when it was reported in birds and horses [24].

These results confirm the coexistence of different genetic clades of WNV lineage 1, along with the recently reported occurrence of WNV lineage 2 [6], documenting a remarkable increase in the diversity of WNV populations in Southern Spain. This increase in the genetic diversity highlights the potential for altered virulence, host range, or transmission dynamics, and underscores the critical importance of proactive, integrated surveillance strategies combining vector monitoring, genomic sequencing, and rapid response measures, as exemplified by the Andalusian Programme for Surveillance and Control of WNV Vectors [12]. Advancing our understanding of the environmental and genomic determinants of WNV transmission remains essential for forecasting outbreaks and guiding targeted interventions. In this context, genomics-informed surveillance platforms, such as SIEGA [8], offer valuable support for real-time detection, lineage tracking, and evidence-based public health decision-making in response to emerging zoonotic threats [26].

The following are the supplementary data related to this article.

Supplementary Table 1 A set of 228 whole genomes of WNV lineage 1 from previous outbreaks found in the GenBank repository. Supplementary Table 1

## CRediT authorship contribution statement

**Carlos S. Casimiro-Soriguer:** Methodology, Investigation. **Maria Lara:** Investigation, Formal analysis, Data curation. **Irene Pedrosa-Corral:** Resources, Methodology. **Cristina Gómez-Camarasa:** Resources. **Nicola Lorusso:** Conceptualization. **J. Alberto Chaves:** Conceptualization. **Jose M. Navarro-Marí:** Resources. **Laura Merino:** Resources. **Jose A. Lepe:** Resources, Methodology. **Joaquin Dopazo:** Writing – review & editing, Writing – original draft, Supervision, Resources, Conceptualization. **Sara Sanbonmatsu-Gámez:** Writing – review & editing, Writing – original draft, Methodology, Investigation, Conceptualization. **Javier Perez-Florido:** Writing – review & editing, Writing – original draft, Supervision, Methodology, Investigation, Formal analysis, Conceptualization.

## Ethical issues

Viral samples were provided and sequenced in the context of the Andalusian Protocol for WNF surveillance and alert [9]. Only epidemiological data disconnected from the affected individual are used in the study, and no ethical approval was required.

## Declaration of competing interest

Joaquin Dopazo reports administrative support, article publishing charges, and statistical analysis were provided by Horizon Europe. Joaquin Dopazo reports administrative support and statistical analysis were provided by Carlos III Health Institute. The other authors declare that they have no known competing financial interests or personal relationships that could have appeared to influence the work reported in this paper.

## Data Availability

The WNV sequences reported are available in the European Nucleotide Archive (ENA) database under the project identifier PRJEB43037
